# Salivary microbiome in peritoneal dialysis patients with and without sarcopenia: A pilot study

**DOI:** 10.1371/journal.pone.0330767

**Published:** 2025-08-22

**Authors:** Wararak Choovanichvong, Sirirak Supa-Amornkul, Dulyapong Rungraungrayabkul, Natcha Boonyapratheeprat, Sasiwimon Meenetkum, Sarinya Boongird, Piyatida Chuengsaman, Chagriya Kitiyakara, Sujiwan Seubbuk Sangkhamanee

**Affiliations:** 1 Department of Oral Medicine and Periodontology, Faculty of Dentistry, Mahidol University, Bangkok, Thailand; 2 Department of Oral Microbiology, Faculty of Dentistry, Mahidol University, Bangkok, Thailand; 3 Division of Nephrology, Department of Medicine, Faculty of Medicine Ramathibodi Hospital, Mahidol University, Bangkok, Thailand; 4 Department of Epidemiology, Faculty of Public Health, Mahidol University, Bangkok, Thailand; 5 Banphaeo-Charoenkrung Peritoneal Dialysis Center, Banphaeo Dialysis Group, Banphaeo Hospital, Bangkok, Thailand; Louisiana State University Health Sciences Center School of Dentistry, UNITED STATES OF AMERICA

## Abstract

**Purpose:**

To investigate salivary microbiota composition in end-stage kidney disease (ESKD) patients with sarcopenia (SESKD), ESKD patients without sarcopenia (NSESKD), and individuals without chronic kidney disease (control group).

**Materials and Methods:**

Thirty-three participants were enrolled: 10 SESKD patients, 12 NSESKD patients, and 11 controls. Demographic data, oral examinations, and unstimulated saliva samples were collected. Salivary bacterial microbiomes were analyzed using high-throughput sequencing targeting the V3-V4 region of the bacterial 16S rRNA gene.

**Results:**

The overall bacterial abundance and distribution were significantly higher in the SESKD and NSESKD groups compared to the control group (*p* < 0.05), with no significant differences between the SESKD and NSESKD groups. ESKD, educational level, and muscle strength were significantly associated with variations in the salivary microbiome composition. Analysis of bacterial abundance revealed a shift in trends as the disease combined from control to NSESKD and SESKD group, respectively, across 7 genera: *Actinobacillus*, *TM7x*, *Capnocytophaga*, *Neisseria*, and *Leptotrichia* increased in abundance, while *Actinomyces* and *Atopobium* decreased. Linear discriminant analysis effect size (LEfSe) identified *Leptotrichia* as a potential biomarker for ESKD (both with and without sarcopenia).

**Conclusion:**

ESKD condition impacted microbial composition, with minimal influence from sarcopenia. Specifically, *Leptotrichia* was notably higher in the ESKD group.

## Introduction

Chronic kidney disease (CKD), characterized by reduced renal function accompanied by multiple comorbidities, is among the top ten causes of death worldwide [1]. The global prevalence of CKD is estimated to be between 11% and 13%, with higher rates in parts of Asia [2,3]. CKD patients may progress to ESKD, requiring long-term kidney replacement therapies such as peritoneal dialysis (PD) or hemodialysis (HD) to remove uremic toxins and maintain fluid and electrolyte balance [1]. Nonetheless, dialysis is insufficient to correct metabolic disturbances, leading to uremic conditions associated with systemic inflammation and immune dysregulation, contributing to functional decline, muscle wasting, and increased cardiovascular risk in CKD [4,5].

Sarcopenia, defined by loss of skeletal muscle mass and function, is typically diagnosed in the elderly population. However, sarcopenia is a widespread complication in CKD, particularly in advanced stages [6–10]. The prevalence of sarcopenia among individuals with CKD varies widely, ranging from 4% to 42%, depending on the specific definition used, the population studied, and the stage of the disease [11]. Several CKD-related factors, including chronic inflammation, physical inactivity, metabolic acidosis, inadequate protein intake, and dialysis-associated protein loss, contribute to muscle degradation and sarcopenia, further increasing the risk for disability, hospitalization, and mortality [12,13].

CKD patients often have poor oral health due to increased ammonia generation from accumulated urea, reduced salivary flow, increased salivary pH [14], inflammation, and compromised immune function [15]. Dysbiosis, or an imbalance in the oral gut microbial ecosystem, especially the gut microbiome, is increasingly recognized in the pathogenesis of CKD and its complications [16]. The oral cavity is second only to the gut regarding the abundance and diversity of microorganisms with distinct microbiome characteristics depending on the specific oral site. The salivary microbiome, representing microbes shed from various oral surfaces, is a valuable indicator of oral microbial integrity. Given that daily saliva production exceeds 1000 mL, which is subsequently swallowed, oral dysbiosis may significantly influence gut microbiota composition, potentially amplifying systemic inflammation [17–19]. Studies have identified alterations in the salivary microbiome among pre-dialysis CKD and hemodialysis patients compared to healthy individuals [20–22]. However, research regarding peritoneal dialysis patients, who experience more stable blood uremic toxin levels due to continuous dialysis treatment, remains limited. Moreover, the relationship between the oral microbiome and CKD clinical complications has not been well-studied.

Oral health and microbiome alterations may have a bidirectional relationship with sarcopenia. In elderly populations without CKD, sarcopenia may impair oral muscle function and nutritional intake and increase periodontal disease prevalence, further enhancing muscle loss [23]. Conversely, oral microbiome dysbiosis may enhance inflammation, infections, and protein-energy wasting, accelerating muscle breakdown [24]. Sarcopenia is associated with reduced gut microbial diversity in non-CKD subjects [25], but data on the relationship between the oral microbiome and sarcopenia remains limited.

Despite increasing interest, the potential relationships between oral microbial ecosystems and sarcopenia, particularly in CKD patients undergoing peritoneal dialysis, are largely unexplored. This pilot study aims to characterize the salivary microbiome of ESKD patients on peritoneal dialysis, comparing individuals with and without sarcopenia and healthy controls. Understanding the role of oral microbiome in CKD-associated sarcopenia may enable early detection and allow integrated oral and medical care plans for these patients.

## Materials and methods

### Population characteristics

This cross-sectional study enrolled three groups of patients aged over 20 years, from May 2023 to December 2024. The first two groups were ESKD patients undergoing chronic peritoneal dialysis for over 3 months at the Banphaeo Dialysis Group in Bangkok, Thailand. The participants were classified into two groups based on the presence or absence of sarcopenia, according to the Asian Working Group for Sarcopenia 2019 guidelines [26]. Patients with a previous kidney transplant were excluded. The third group of participants was non-CKD controls, which were defined as individuals who were healthy or had controlled systemic diseases but had no diagnosis of CKD. These controls were selected based on age and sex and matched with the ESKD group. The exclusion criteria for all groups were advanced chronic diseases such as cirrhosis and cancer, recent antibiotics within the past three months, and a positive COVID-19 test.

This study was conducted according to the Declaration of Helsinki with approval from the ethics committees of the Institutional Review Board, Faculty of Medicine, Ramathibodi Hospital (MURA 2021/218 and 2024/692). All individuals signed a written informed consent.

### Demographic data, physical and oral examination, and sarcopenia assessment

A trained research team obtained demographic and examination data using a standardized protocol and questionnaire. The patients’ weight and height were measured using a bioelectrical impedance analysis (BIA) using an 8-electrode multifrequency device (InBody 770, InBody Co., Ltd. Seoul, South Korea) and a stadiometer, respectively. For participants in the ESKD group, dry weight values were recorded. All participants were instructed to remove their shoes and wear light clothing during the measurements.

### Sarcopenia assessment

Sarcopenia was defined by the Asian Working Group for Sarcopenia (AWGS) 2019, which is considered standard criteria for Asian subjects as “loss of skeletal muscle mass, plus loss of muscle strength, and/or reduced physical performance” [26].

Appendicular skeletal muscle mass (ASM) was quantified by BIA using an 8-electrode multifrequency device (InBody 770, InBody Co., Ltd. Seoul, South Korea). *Low muscle mass* was defined as ASM/height^2^: men < 7.0 kg/m^2^, women < 5.7 kg/m^2^.

Muscle strength was assessed by handgrip strength using the Digital Grip Strength Dynamometer (T.K.K 5401, Takei Scientific Instruments Co., Ltd., Tokyo, Japan). *Low muscular strength* was defined as hand grip: men < 28 kg, women < 18 kg.

The physical performance was assessed using the chair stand test. With arms folded across their chest, participants were instructed to stand up and sit down as quickly as possible for five repetitions. A completion time of ≥ 12 seconds indicated *reduced physical performance*.

### Oral health assessment

For the oral examination, several indices were utilized: the simplified oral hygiene index (OHI-S) for a quantitative assessment of oral hygiene [27], the modified gingival index (MGI) for the early detection of clinical signs of gingival inflammation [28], as well as evaluations of tooth mobility, the number of teeth (NRT) [29], and the count of posterior occluding pairs (POPs) [30].

### Saliva collection

Saliva collection was conducted in the morning, with participants instructed to refrain from consuming food or beverages for at least two hours prior to the procedure. Each participant provided a 5-ml saliva sample into a 50 mL conical sterile polypropylene tube by spitting method [31] to measure the unstimulated salivary flow rate. The flow rate was recorded. A complete™, Mini protease inhibitor cocktail (Roche Diagnostics GmbH, Germany) was subsequently added to the saliva sample, which was then centrifuged at 10,000 g at 4°C for 10 minutes. The resulting supernatant was aliquoted and promptly stored at −80°C until used.

### DNA extraction and 16s rDNA sequencing

A 100-microliter saliva sample was thawed at room temperature and homogenized using a vortex. DNA extraction was carried out using the QIAamp® DNA MiniKit (Qiagen, Hilden, Germany). The extracted DNA samples were subsequently submitted to Vishuo Biomedical for 16S rDNA amplicon sequencing. The sequencing library was constructed utilizing the MetaVX Library Preparation Kit (Genewiz, South Plainfield, NJ, USA). In brief, 20–50 ng of DNA was used to generate amplicons that cover V3 and V4 hypervariable regions of the 16s rRNA gene of bacteria. The forward primers contain the sequence ‘ACTCCTACGGGAGGCAGCAG’ and the reverse primers contain the sequence ‘GGACTACHVGGGTWTCTAAT’. At the same time, indexed adapters will be added to the ends of the 16S rDNA amplicons to generate indexed libraries. DNA libraries were multiplexed and loaded onto an Illumina MiSeq/Novaseq platform (Illumina, San Diego, USA) for sequencing. Automated cluster generation and 250/300 paired-end sequencing with dual reads were conducted in accordance with the manufacturer’s protocols. Raw 16s rRNA amplicon sequencing data are available with accession bioproject number PRJNA1249468.

### Data analysis

Sequencing analysis was conducted using QIIME2 (version 2019.4.0) [32]. Double-end sequencing reads, including both positive and negative reads, were processed through DADA2 for filtering, denoising, and chimera removal to obtain amplified sequence variants (ASVs). Then, RDP Classifier (Ribosomal Database Program) Bayez algorithm was used to compare ASV representative sequences with Silva_138 16S database, thereby to obtain the community composition of each sample. Alpha (Chao1, Shannon) and beta (principal coordinates analysis: PCoA, analysis of similarities: ANOSIM) diversity indices were determined by using QIIME2. Potential bacterial genera biomarkers for each group were identified through LEfSe and linear discriminant analysis (LDA). An LDA score greater than 3 was considered to indicate a significant difference between groups. Additionally, the significant factors among groups were further analyzed for their potential impact on the bacterial community structure in addition to the condition of ESKD and sarcopenia.

### Statistical analysis

Continuous Data is shown as mean ± SD or median IQR for normally and non-normally distributed data respectively. Categorical data was shown as n (%) and groups were compared using Analysis of Variance (ANOVA) or Fisher’s exact test as appropriate. Statistical analysis was performed using a software package (IBM SPSS Statistics version 29). Statistical significance was considered when the *p*-value was less than 0.05.

## Results

### Demographic, physical, and oral characteristic

The demographic data and physical and oral examination of all participants were summarized in Table 1.

**Table 1:** Demographic data, oral status and physical examination data.

Factors	ESKD with Sarcopenic (n = 10)	ESKD with non-Sarcopenic (n = 12)	Control (n = 11)	*p*-Value
**Demographic data**				
**Age (years) [Mean ± SD]**	64.70 ± 11.6	64.67 ± 11.0	64.36 ± 11.5	0.997^†^
**Genders [n (%)]**				
Male	6 (60.0)	8 (66.7)	7 (63.6)	1.000^‖^
Female	4 (40.0)	4 (33.3)	4 (36.4)	
**BMI [Mean ± SD]**	20.20 ± 3.9^ab^	24.76 ± 2.8^a^	25.34 ± 3.9^b^	0.004^†^
**Socioeconomic status**				
Low (≤15,000 baht/month)	6 (60.0) ^a^	12 (100.0)^a b^	4 (36.4)^b^	0.002^‖^
Middle/High (>15,000 baht/month)	4 (40.0)	(0.0)	7 (63.6)	
**Educational level [n (%)]**				
≤Primary school	6 (60.0)^a^	9 (75.0)^b^	1 (9.1)^ab^	0.004^‖^
≥Middle school	4 (40.0)	3 (25.0)	10 (90.1)	
**Alcohol consumption [n (%)]**				
No	10 (100.0)	11 (91.7)	7 (63.6)	0.057^‖^
Occasionally/Regularly	(0.0)	1 (8.3)	4 (36.4)	
**Smoking [n (%)]**				
No	6 (60.0)	8 (66.7)	9 (81.8)	0.575^‖^
>100 cigarettes/life time	4 (40.0)	4 (33.3)	2 (18.2)	
**Dental visit/year [n (%)]**				
Never	7 (70.0)^a^	10 (83.3)^b^	1 (9.1)^ab^	0.001^‖^
Yes	3 (30.0)	2 (16.7)	10 (90.1)	
**Oral status**				
**Xerostomia [n (%)]**				
No	4 (40.0)^a^	4 (33.3)^b^	10 (90.1)^ab^	0.010^‖^
Yes	6 (60.0)	8 (66.7)	1 (9.1)	
**Salivary flow rate; mL/min [Median (IQR)]**	0.37 (0.22,0.56)	0.29 (0.16,0.45)	0.56 (0.25,0.92)	0.12^‖^
**DI-S [Median (IQR)]**	1.09 (1.00,1.85)	1.75 (1.23,2.00)^a^	1 (0.75,1.00)^a^	0.003^‖^
**CI-S [Median (IQR)]**	0.83 (0.67,1.00)	1.08 (0.87,1.57)^a^	0.4 (0.00,0.83)^a^	0.010^‖^
**OHI-S [Median (IQR)]**	2.00 (1.67,2.95)	2.83 (2.22,3.90)^a^	1.4 (1.00,1.67)^a^	0.002^‖^
**MGI [Median (IQR)]**	2.1 (1.64,2.53)^a^	1.8 (1.2,2.8)^b^	1.2 (0.55,1.45)^ab^	0.007^‖^
**NRT [n (%)]**				
≥ 20 teeth	5 (50.0)^a^	6 (50.0)^b^	11 (100.0)^ab^	0.009^‖^
< 20 teeth	5 (50.0)	6 (50.0)	0 (0.0)	
**POPs** **[n (%)]**				
0 pair	2 (20.0)^a^	3 (25.0)^b^	0 (0.0)^ab^	0.002^‖^
1-7 pairs	8 (80.0)	9 (75.0)	5 (45.5)	
8 pairs	0 (0.0)	0 (0.0)	6 (54.5)	
**Physical examination**				
**BIA [n (%)]**				
Normal	0 (0.0)^ab^	12 (100.0)^b^	9 (81.8)^a^	0.000^‖^
Under cutoff point (M: < 7.0 kg/m^2^, F: < 5.7 kg/m^2^)	10 (100.0)	0 (0.0)	2 (18.2)	
**Handgrip strength [n (%)]**				
Normal	1 (10.0)^a^	5 (41.7)^b^	11 (100.0)^ab^	0.000^‖^
Under cutoff point (M: < 28 kg, F: < 18 kg)	9 (90.0)	7 (58.3)	0 (0.0)	
**Chair stand test [n (%)]**				
Normal	4 (40.0)	5 (41.7)	9 (81.8)	0.083^‖^
Beyond cutoff point (≥ 12 s)	6 (60.0)	7 (58.3)	2 (18.2)	

The letters “a” and “b” represent significant differences between the groups (**p <* *0.05).

† One-way ANOVA

‖ Fisher’s exact test

IQR: Interquartile range; BMI: Body mass index; DI-S: Simplified Debris Index; CI-S: Simplified Calculus Index; OHI-S: Simplified Oral Hygiene Index; MGI: Modified gingival index; NRT: Number of teeth; POPs: Posterior Occluding Pairs; BIA: Bioelectrical impedance analysis.

A total of 33 participants were enrolled in the study. Overall, the average age was 64.58 ± 11.01 years, with no differences between groups (**p* *= 0.997). Based on the diagnosis of sarcopenia and dialysis status, the participants were categorized into three groups: 1) SESKD (n = 10), 2) NSESKD (n = 12), and 3) control group (n = 11). BMI in the SESKD group was lower than NSESKD and control groups (*p* = 0.004). The control group reported a greater proportion with higher income and educational levels than those in the SESKD and NSESKD groups (*p* = 0.002, *p* = 0.004, respectively). No differences were observed in alcohol consumption (*p* = 0.057) or smoking status (*p* = 0.575) among the three groups. ESKD patients with and without sarcopenia reported not visiting a dentist throughout the year, whereas 90% of control participants maintained regular dental checkups (*p* = 0.001). The ESKD groups reported a higher frequency of xerostomia and tended to have a lower salivary flow rate than the control group (*p* = 0.010, *p* = 0.12, respectively). According to the simplified oral hygiene index, the average oral hygiene score across all groups was classified as fair (1.3–3.0), with the lowest scores observed in the control were significantly lower than in NSESKD groups (*p* = 0.001). There was a significant difference in gingival inflammation across the groups, with the highest level of inflammation observed in the SESKD group, followed by the NSESKD group and the control group (*p* = 0.007). The number of POPs, which serves as strong indicators of masticatory capacity, was significantly lower in the SESKD and NSESKD groups than in the control group (*p* = 0.002). Regarding sarcopenia parameters, all SESKD participants, none from the NSESKD group and 18.2% of the control group exhibited *low muscle mass* (*p* = 0.000). Additionally, 90% of SESKD participants and 58.3% of NSESKD participants had *low muscle strength* (*p* = 0.000), whereas all control subjects demonstrated normal muscle strength. No significant differences in physical performance were observed across the groups (*p* = 0.083).

### Global sequencing data

Sequencing quality of sequencing data are expressed in Q Phred. At Q20(%), the present study obtained an average of 97.69% ranged from 97.41 to 98.16. A total of 1,445,784 high-quality sequences were generated from 33 salivary samples with an average of 43,811.64 sequences per sample ranged from 33,338–68,181. Total raw data sequence was presented in S1 Table. High-quality sequences were clustered into 2,679 ASV ranged from 70 to 426 per sample corresponding to 318 species, 155 genera, 86 families, 56 orders, 22 classes, and 15 phyla.

### Bacterial abundance and distribution

**Fig 1:**
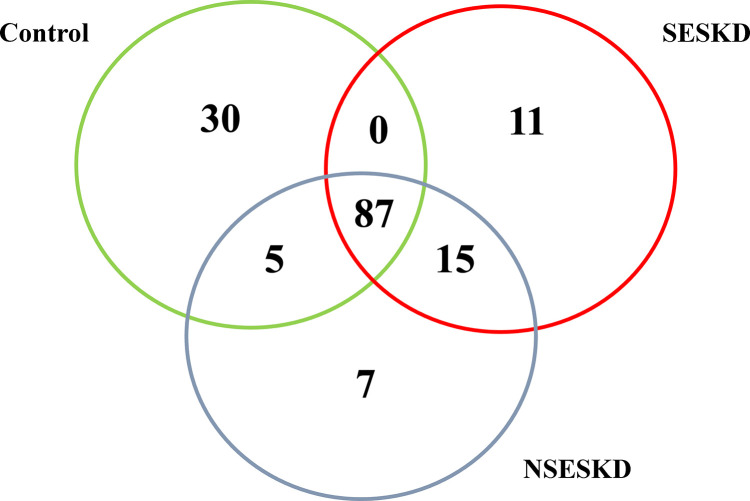
Venn Diagram showing a number of bacterial genera identified among three groups.

**Fig 2:**
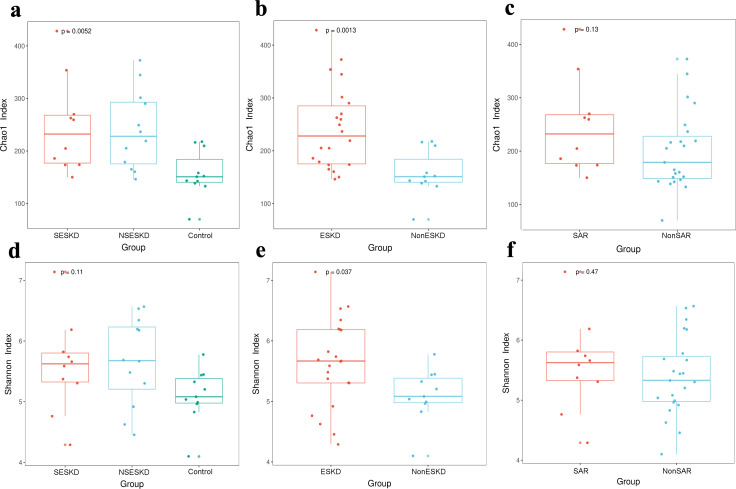
Alpha-diversity indices. Chao1 index (a) between ESKD with sarcopenia (SESKD), ESKD without sarcopenia (NSESKD), and control, (b) between end-stage kidney disease (ESKD) and control (NonESKD), (c) between sarcopenia and non-sarcopenia group. Shannon index (d) between ESKD and sarcopenia (SESKD), ESKD without sarcopenia (NSESKD), and control, (e) between end-stage kidney disease (ESKD) and control (NonESKD), (f) between sarcopenia and non-sarcopenia group. Kruskal–Wallis test, *p < 0.05.

At the genus level, a total of 155 bacterial genera were identified. A total of 87 genera were found to be common across the three groups. Additionally, 11 genera were found exclusively in the SESKD group, 7 were unique to the NSESKD group, and 30 were present solely in the control group (Fig 1). Table 2 displays the bacterial abundance in each group according to different taxonomic classifications. Alpha diversity indices were determined to investigate species diversity across different groups. The Chao1 index was used to evaluate within-sample species richness, while the Shannon index was employed to assess species evenness. The alpha diversity results, including the Chao1 and Shannon indices, are presented in Fig 2. Both indices were significantly higher in the ESKD groups (SESKD, NSESKD) compared to the control group (Kruskal-Wallis test, **p* *< 0.05). The increased values indicate greater bacterial abundance and evenness. No significant differences were observed between the sarcopenia and non-sarcopenia samples (*p* = 0.13, Chao1; *p* = 0.47, Shannon). These findings indicated significant dysbiosis in microbial diversity within the SESKD and NSESKD groups when compared to the control group.

**Table 2:** Bacterial abundance in each group based on different taxonomic classifications.

Taxon	Total Abundance	Bacterial Abundance		
		SESKD	NSESKD	Control
**Phylum**	15	12	13	13
**Class**	22	18	18	18
**Order**	56	45	42	43
**Family**	86	67	64	71
**Genus**	155	113	114	122
**Species**	318	230	231	220

### Bacterial community structure

**Fig 3:**
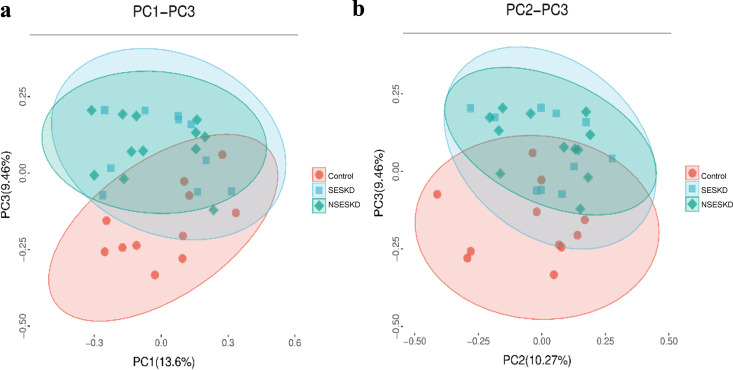
Principal coordinates analysis (PCoA).

Between-group differences were further assessed using PCoA based on unweighted UniFrac distance matrices and ANOSIM. Additionally, various factors related to the subject have been analyzed to determine whether they may influence differences in the microbiome. The beta diversity of the salivary microbiota in patients with ESKD, both with and without sarcopenia, was distinctly separated from that of the control subjects. In contrast, sarcopenia status (SESKD and NSESKD) exhibited poor separation in the PCoA (Fig 3). It is suggested that the structures of bacterial communities in ESKD with and without sarcopenia were similar. In addition, ANOSIM analysis revealed a significant difference in the microbiota diversity in the ESKD group (with and without sarcopenia) compared to the control group (qualitative ANOSIM, R = 0.161, *p* = 0.016). Among the factors associated with the subject, aside from ESKD, the results indicated that educational level and muscle strength were correlated with variations in the salivary microbiome. In contrast, as examined in the present study, xerostomia, oral hygiene index (DI-S, CI-S, and OHI-S), and oral health status (GI, NRT, and POPs) did not demonstrate a significant impact on the microbiome.

**Fig 4:**
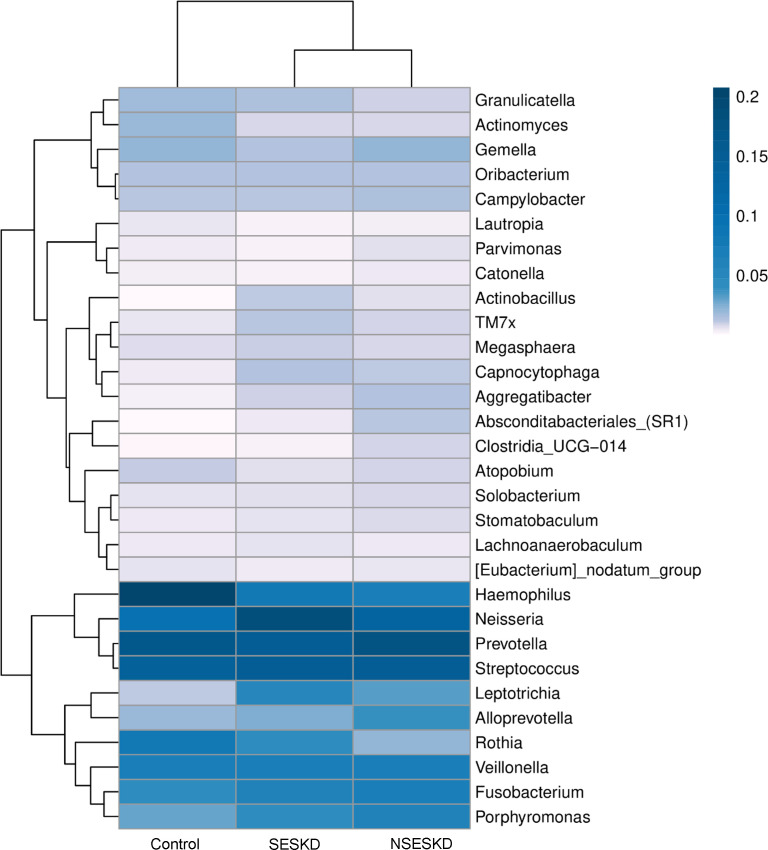
Heatmap demonstrating the relative abundance of genera in each group.

The top 30 genus distribution of each group was clustered and plotted in a heatmap as shown in Fig 4. NSESKD and SESKD exhibited a higher degree of similarity when compared to the control group. A trend in bacterial abundance was observed across 8 genera, with either an increase or decrease in abundance, and differences were noted in an ordinal manner between the control, NSESKD, and SESKD groups. *Actinobacillus, TM7x, Capnocytophaga, Neisseria,* and *Leptotrichia* increased in abundance as the diseases combined, with the highest levels found in the ESKD with sarcopenia group. Conversely, *Actinomyces* and *Atopobium* exhibited a decrease in abundance as the disease state advanced. The analysis of educational level and muscle strength revealed corresponding trends in bacterial abundance, with *Actinobacillus*, *TM7x*, *Capnocytophaga*, *Neisseria*, and *Leptotrichia* showing increased abundance in the low educational level/muscle strength group, while *Actinomyces* and *Atopobium* exhibited decreased abundance.

### Differential microbiota compositions

**Fig 5:**
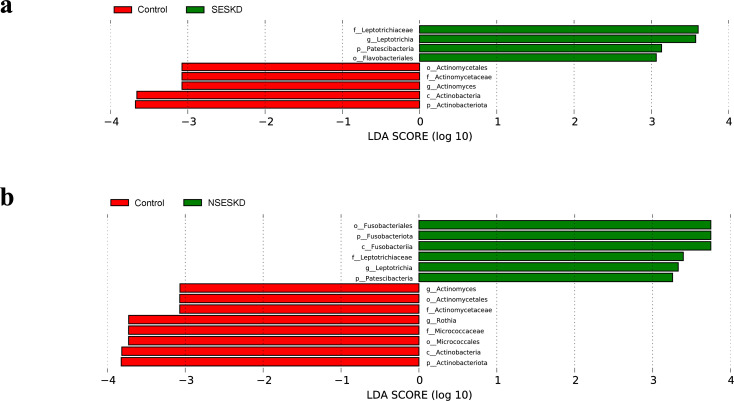
Linear discriminant analysis effect size (LEfSe) analysis. **(a)** ESKD with sarcopenia (SESKD) comparing with controls, **(b)** ESKD without sarcopenia (NSESKD) comparing with controls.

The differentially abundant taxa were subsequently analyzed as potential taxonomic biomarkers of sarcopenia using LEfSe (LDA ≥ 3; Fig 5). This analysis identified the bacterial genus *Leptotrichia* as a potential biomarker for ESKD, both with and without sarcopenia, while *Actinomyces* was identified as a biomarker for the control groups. The ratio of *Leptotrichia* to *Actinomyces* was calculated across three groups: control, SESKD, and NSESKD. The resulting median ratios were as follows: 0.47 (IQR = 0.3, 1.47) for the control group, 4.45 (IQR = 1.55, 7.31) for the NESKD group and 3.18 (IQR = 2.19, 6.97) for the SESKD group. Notably, a significant difference was observed in the *Leptotrichia*/*Actinomyces* ratio in saliva samples between the ESKD and control groups (*p* = 0.001), while no significant difference was found between the ESKD groups with and without sarcopenia.

## Discussion

Saliva collection is a noninvasive, rapid, cost-effective, and simple method for monitoring oral microbiome. Saliva captures microbial communities from various oral niches, including the tongue and buccal mucosa, thus providing a broader representation of the oral microbiome compared to dental plaque, which forms a more isolated biofilm environment with reduced diversity and abundance [33]. In this study, we characterized the salivary microbiome in ESKD patients undergoing peritoneal dialysis, comparing those with and without sarcopenia to controls. The major finding was that the overall taxonomic diversity, as indicated by alpha-diversity indices in richness and evenness, was higher in individuals with ESKD, with no significant differences between those with and without sarcopenia. Beta-diversity analysis showed significant associations between ESKD, educational level, and muscle strength, whereas other demographic factors and oral health parameters showed minimal impact.

Saliva usually maintains a warm environment with a pH ranging from 6.75 to 7.25, which is crucial in modulating the oral microbiome [34]. Several pathophysiological factors in CKD patients may alter the salivary environment, contributing to observed microbiome changes. Reduced salivary flow and elevated salivary pH, frequently observed in advanced CKD, influence microbial composition [14,35]. Impaired renal excretion increases serum and salivary urea and creatinine concentrations, promoting ammonia production and raising oral pH, affecting microbial communities [36–38]. Uremia can influence the intestinal microbiota’s composition and compromise the intestinal barrier’s integrity. This disruption increases the translocation of bacterial products across the intestinal barrier, activating innate immunity and inducing systemic inflammation [15]. Systemic inflammation may further disrupt the composition and balance of the oral microbiota, as has been reported in several diseases [39,40]. Patients with ESKD on hemodialysis and peritoneal dialysis have distinct characteristics that may contribute to changes in the human microbiome. These include specific dietary restrictions, different types of dialysis access (vascular vs. peritoneal), and the continuous inflow of peritoneal dialysate leading to glucose absorption and a more stable removal of uremic toxin versus swings in toxin levels due to the intermittent nature of hemodialysis [41]. To date, there are limited studies of oral microbiome in peritoneal dialysis patients. Our finding of increased alpha diversity in peritoneal dialysis patients aligns with prior studies in pre-dialysis CKD and ESKD patients treated by hemodialysis, although results have varied [21,22,42]. In hemodialysis patients, Duan et al. (2020) found that HD patients exhibited lower salivary microbial richness but higher evenness [21], while Gan et al. (2023) reported an increase in both salivary microbial richness and evenness in HD patients [22]. Additionally, Guo et al. (2022) examined oral microbial diversity in pre-dialysis CKD patients using pharyngeal swabs, demonstrating an increase in microbial diversity in the oral cavity of CKD patients compared to healthy controls [42]. Whereas Liu et al. (2022) found a distinct salivary microbiome in pre-dialysis CKD patients compared to healthy controls, regardless of co-occurrence diseases of diabetes and hypertension. However, no differences in bacterial richness and diversity were observed between the two groups [20].

Our Beta-diversity analyses revealed that ESKD, educational level, and muscle strength were significantly associated with variations in the salivary microbiome across the groups. At the same time, other demographic factors and oral health status did not show significant differences. The results of the present study show that patients with ESKD, both with and without sarcopenia, had poorer oral health compared to the control group. This was demonstrated by significantly higher oral hygiene index scores, more gingival inflammation, and fewer remaining teeth, suggesting a greater history of dental caries and periodontal disease in the ESKD groups. Although the salivary microbiome can reflect local bacterial changes in the supragingival and subgingival microbiota, and features associated with periodontitis and dental caries have been identified, most prior studies have relied on stimulated saliva samples for microbiome analysis [43]. In contrast, the present study utilized unstimulated saliva collected via the spitting method. The comparability of oral microbiome profiles between stimulated and unstimulated saliva remains controversial [44,45], which may partly explain why no significant differences in salivary microbiome composition were observed across the study groups, despite the ESKD patients’ tendency toward poor oral health. Notably, a recent systematic review and meta-analysis reported that red-complex bacteria, key pathogens in periodontitis, are found at significantly lower levels in saliva compared to subgingival plaque in periodontitis patients, in both detection frequency and relative abundance [46]. From an oral health assessment perspective, dental plaque may therefore be a more appropriate sample type than saliva. However, it is important to note that the primary aim of the present study was not to assess oral health status; but rather to compare the salivary microbiome among individuals with ESKD, both with and without sarcopenia, and healthy controls. The lack of association between the salivary microbiome and both oral hygiene index and gingival inflammation supports a systemic, rather than local, effect on the salivary microbiome. Our findings that the bacterial community structure also showed significant differences with educational levels is consistent with a previous study from Hallang et al. (2021), who reported that a low educational level was associated with an unfavorable microbiota composition in Swedish volunteers, regardless of CKD and other systemic conditions [47].

At the genus level, an increase in the abundance of *Actinobacillus*, *TM7x*, *Capnocytophaga*, *Neisseria*, and *Leptotrichia* was observed in the ESKD group. In contrast, *Actinomyces* and *Atopobium* exhibited a decline in abundance as the diseases combined. The result corresponded to previous studies [20–22,48], except for *Actinobacillus*, as Gan et al. (2023) reported, which showed an decrease in abundance in hemodialysis patients. These certain bacteria are commensal members of healthy human oral communities. However, under certain conditions, these bacteria can act as opportunistic pathogens, contributing to the development of various diseases. LEfSe analysis suggested *Leptotrichia* and *Actinomyces* as potential biomarker for ESKD and control, respectively. *Leptotrichia* can act as opportunistic pathogens, contributing to a variety of diseases, particularly in immunocompromised individuals, though they may also affect immunocompetent hosts [49]. Like other Gram-negative rods, *Leptotrichia* contains lipopolysaccharide (LPS, endotoxin), which stimulates the immune system. The specific immune response triggered by *Leptotrichia* depends on the abundance of various species clusters. Within the *Prevotella* cluster, which includes potentially pathogenic species, *Leptotrichia* has been shown to increase the transcription levels of proinflammatory cytokines such as interleukin (IL)-1β, IL-6, IL-8, and IL-10 in epithelial cells. This suggests that *Leptotrichia* may play a crucial role in the transition from health to disease. The data strongly support the idea that *Leptotrichia* is significantly involved in the fine-tuned regulation of epithelial immune responses, contributing either to maintaining immune homeostasis or propagating inflammatory responses [50]. In addition, the *Actinomyces* genus, proposed as a potential biomarker for control group, are Gram-positive bacteria that are commensal members of the human oral cavity. *Actinomyces* are core salivary microbiome of the healthy microbiome, with a relative abundance greater than 1%, but their levels are significantly reduced in the ESKD community [21]. Most *Actinomyces* infections are polymicrobial, and current literature provides limited information on the virulence properties of *Actinomyces* species. Aside from their production of urease and biofilms, *Actinomyces* do not typically express classical virulence factors [51]. *Actinomyces naeslundii* is an important ureolytic organism in the oral cavity. Urease activity in *A. naeslundii* was highest at pH 6.0 and decreased as the pH increased [52]. It is possible that in higher pH conditions, such as those found in the salivary samples of ESKD patients, urease activity, along with the presence of *Actinomyces*, may decline.

Emerging evidence suggests that gut microbiota dysbiosis plays an important role in the development of sarcopenia by influencing systemic inflammation, nutrient absorption, and muscle metabolism in both CKD and non-CKD populations with findings indicating an overall reduction in microbial diversity in sarcopenic individuals [25,53–55]. Oral microbiome dysbiosis may similarly exacerbate systemic inflammation and contribute to muscle wasting by promoting bacterial translocation and inflammatory mediator production directly or indirectly by modifying the gut microbiome [17–19]. To our knowledge, our study is the first to investigate the role of the oral microbiome in sarcopenia. While there was some evidence that beta diversity was linked to muscle strength, we did not find significant differences in alpha diversity or other parameters based on sarcopenia status. The lack of significant difference in this pilot study may reflect the relatively small sample size, the subtle nature of microbiome-related differences in sarcopenia, or the masking effects of dialysis and systemic conditions common to both sarcopenic and non-sarcopenic ESKD patients.

Our study has the following implications. First, salivary microbiome composition could distinguish peritoneal dialysis patients from healthy controls. This reinforces the potential role of oral dysbiosis in the pathogenesis of ESKD and its complications. The similar trend of our novel findings in the peritoneal dialysis patients with previous studies in hemodialysis patients is consistent with a dominant systemic effect of uremia on oral salivary microbiome profiles despite patients being on dialysis. Future research should focus on validating salivary microbiome biomarkers identified here, such as *Leptotrichia*, and explore therapeutic interventions targeting oral microbiota to potentially mitigate systemic complications associated with CKD. Finally, peritoneal dialysis patients had worse oral health status than controls, emphasizing the need for a better understanding of the pathogenesis of oral abnormalities in ESKD and the limitations patients face in accessing public health care. This supports the role of integrated dental and medical care approaches to managing CKD complications effectively.

This study has several limitations. Firstly, the small sample size may have limited the statistical power to detect subtle microbiome differences between sarcopenic and non-sarcopenic patients. Additionally, the cross-sectional design restricts the ability to establish causality between microbiome alterations and ESKD development. Potential confounding factors such as dietary habits, medication use, co-morbid diseases, and lifestyle differences were not fully controlled and could influence microbiome composition. Lastly, our study focused solely on saliva samples; future studies incorporating dental plaque and gut microbiota analyses could provide a more complete understanding of microbiome alterations specific to oral health status in ESKD and their implications for sarcopenia.

## Conclusions

This study identified significant differences in the salivary microbiome between ESKD patients undergoing peritoneal dialysis and healthy controls, reinforcing the potential role of oral microbiota in CKD pathogenesis. Although sarcopenia exhibited minimal additional impact on microbial composition, oral dysbiosis may contribute to CKD complications through mechanisms such as systemic inflammation. Further research with larger cohorts is necessary to understand the interactions between oral and gut microbiomes in CKD and sarcopenia. Eventually, microbiota-based interventions could emerge as potential strategies to prevent complications associated with ESKD.

## Supporting information

S1 Table Sequences data per sample. (DOCX)
