# Supplementary material for: Salivary microbiome in peritoneal dialysis patients with and without sarcopenia: A pilot study
Source: PLoS One. 2025 Aug 22;20(8):e0330767. doi: 10.1371/journal.pone.0330767 (PMC12373162; doi:10.1371/journal.pone.0330767)
Supplement: S1 Table — (DOCX) [file pone.0330767.s001.docx]

**S Table. Sequences data per sample.**

| **Sample** | **Length (bp)** | **#Reads** | **Bases (bp)** |
| --- | --- | --- | --- |
| SESKD 1 | 250 | 126944 | 31736000 |
| SESKD 2 | 250 | 131244 | 32811000 |
| SESKD 3 | 250 | 101808 | 25452000 |
| SESKD 4 | 250 | 136328 | 34082000 |
| SESKD 5 | 250 | 149906 | 37476500 |
| SESKD 6 | 250 | 184602 | 46150500 |
| SESKD 7 | 250 | 189298 | 47324500 |
| SESKD 8 | 250 | 162318 | 40579500 |
| SESKD 9 | 250 | 166256 | 41564000 |
| SESKD 10 | 250 | 159792 | 39948000 |
| NSESKD 1 | 250 | 150174 | 37543500 |
| NSESKD 2 | 250 | 175084 | 43771000 |
| NSESKD 3 | 250 | 183932 | 45983000 |
| NSESKD 4 | 250 | 107818 | 26954500 |
| NSESKD 5 | 250 | 129754 | 32438500 |
| NSESKD 6 | 250 | 129908 | 32477000 |
| NSESKD 7 | 250 | 150552 | 37638000 |
| NSESKD 8 | 250 | 146508 | 36627000 |
| NSESKD 9 | 250 | 159620 | 39905000 |
| NSESKD 10 | 250 | 132584 | 33146000 |
| NSESKD 11 | 250 | 159042 | 39760500 |
| NSESKD 12 | 250 | 117412 | 29353000 |
| Control 1 | 250 | 132674 | 33168500 |
| Control 2 | 250 | 115910 | 28977500 |
| Control 3 | 250 | 101414 | 25353500 |
| Control 4 | 250 | 110844 | 27711000 |
| Control 5 | 250 | 115890 | 28972500 |
| Control 6 | 250 | 120914 | 30228500 |
| Control 7 | 250 | 100572 | 25143000 |
| Control 8 | 250 | 182404 | 45601000 |
| Control 9 | 250 | 112248 | 28062000 |
| Control 10 | 250 | 124210 | 31052500 |
| Control 11 | 250 | 117416 | 29354000 |
